# Photocaging of Pyridinylimidazole-Based Covalent JNK3 Inhibitors Affords Spatiotemporal Control of the Binding Affinity in Live Cells

**DOI:** 10.3390/ph16020264

**Published:** 2023-02-09

**Authors:** Beate Sandra Hoffelner, Stanislav Andreev, Nicole Plank, Pierre Koch

**Affiliations:** Department of Pharmaceutical/Medicinal Chemistry II, Institute of Pharmacy, University of Regensburg, Universitätsstraße 31, 93053 Regensburg, Germany

**Keywords:** kinase inhibitor, pyridinyl imidazole, covalent inhibitor, photoactivation, caging group, JNK3

## Abstract

The concept of photocaging represents a promising approach to acquire spatiotemporal control over molecular bioactivity. To apply this strategy to pyridinylimidazole-based covalent JNK3 inhibitors, we used acrylamido-*N*-(4-((4-(4-(4-fluorophenyl)-1-methyl-2-(methylthio)-1*H*-imidazol-5-yl)pyridin-2-yl)amino)phenyl)benzamide (**1**) as a lead compound to design novel covalent inhibitors of JNK3 by modifying the amide bond moiety in the linker. The newly synthesized inhibitors demonstrated IC_50_ values in the low double-digit nanomolar range in a radiometric kinase assay. They were further characterized in a NanoBRET^TM^ intracellular JNK3 assay, where covalent engagement of the target enzyme was confirmed by compound washout experiments and a loss in binding affinity for a newly generated JNK3(C154A)-NLuc mutant. The most potent compound of the series, *N*-(3-acrylamidophenyl)-4-((4-(4-(4-fluorophenyl)-1-methyl-2-(methylthio)-1*H*-imidazol-5-yl)pyridin-2-yl)amino)benzamide (**13**), was equipped with a photolabile protecting group leading to a nearly 10-fold decrease in intracellular JNK3 binding affinity, which was fully recovered by UV irradiation at a wavelength of 365 nm within 8 min. Our results highlight that photocaged covalent inhibitors can serve as a pharmacological tool to control JNK3 activity in live cells with light.

## 1. Introduction

The family of c-Jun N-terminal kinases (JNKs) consists of three kinases, namely JNK1, JNK2, and JNK3. The JNKs are serine threonine kinases which differ in their distribution pattern. In particular, JNK1 and JNK2 are ubiquitously expressed, while JNK3 is expressed predominantly in neuronal tissue and, to a lesser degree, in cardiac myocytes and testis [1,2,3]. Due to its unique expression pattern, JNK3 is a promising target for the treatment of various neurodegenerative disorders, such as Alzheimer’s disease, Parkinson’s disease, and Huntington’s disease [4,5,6]. 

**Figure 1:**
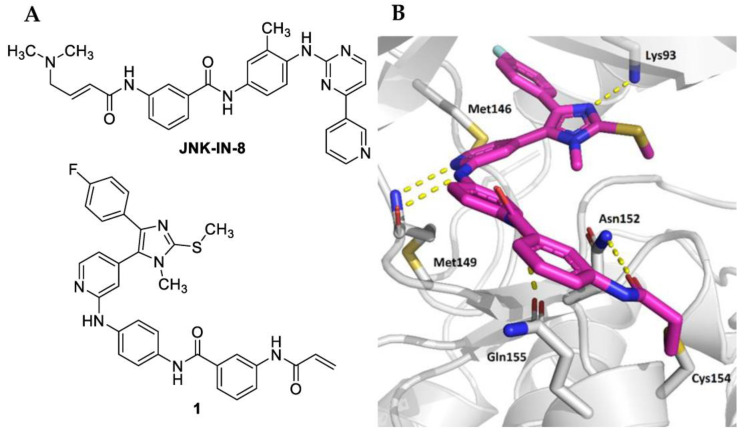
(**A**) Structures of covalent pan-JNK inhibitors **JNK-IN-8** and **1**. (**B**) Crystal structure of compound **1** in complex with JNK3 (PDB code—7ORF). Polar contacts are indicated by yellow dashed lines.

The amino acid sequence within the JNK family is highly conserved, and all three isoforms harbor a cysteine in position 154 (JNK3 numbering), which is unique within the kinome [7]. Two series of selective covalent pan-JNK inhibitors addressing this cysteine side chain with electrophilic Michael acceptor systems have been reported so far. The first series of covalent JNK3 inhibitors was disclosed by Gray and coworkers by modifying an Imatinib-derived lead structure in 2012. **JNK-IN-8** represents the most promising covalent pan-JNK inhibitor of this series (Figure 1A) [8]. In 2017, some of us reported a second series of covalent JNK3 inhibitors based on a pyridinylimidazole scaffold, including compound **1** as the most potent and most selective inhibitor within this series (Figure 1A) [9]. Recently, the binding mode of **1** within the ATP binding site was resolved [10]. As shown in Figure 1B, compound **1** interacts with JNK3 via a bidentate hydrogen bond between the 2-aminopyridine moiety and Met149, and the imidazole ring forms an essential interaction with Lys93. The 4-fluorophenyl moiety is located in the hydrophobic region I. A *para*-*meta*-substitution pattern regarding the two benzene rings of the linker affords the highest target affinity and allows the acrylamide warhead to move into an optimal position for covalent bond formation with JNK3. Moreover, this substitution pattern also allows the linker to interact with Asn152 and Gln155 with its two amide moieties.

Photochemical reactions allow for precise spatiotemporal control of chemical and biological processes as well as bioactive compounds [11,12]. Recently, Wu et al. highlighted photochemical approaches as a promising strategy to minimize off-target binding, especially regarding anti-cancer drugs [11].

**Figure 2:**
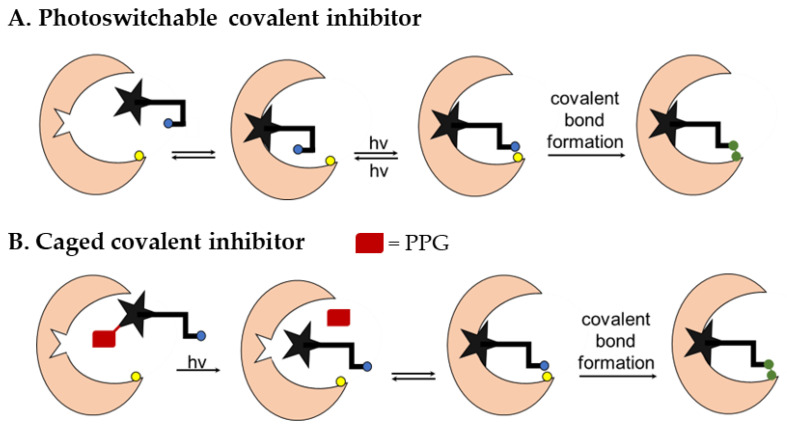
Principles of photoswitching (**A**) and photocaging (**B**) in the case of covalent inhibitors.

The two most prominent approaches for applying photochemical reactivity to bioactive compounds are photoswitching and photocaging [11,13]. Photoswitching refers to a method where a molecule can be present in a *trans* or *cis* configuration that can be interconverted by light pulses of a specific wavelength. In the case of covalent kinase inhibitors targeting non-catalytic cysteines, one of the two configurations orients the electrophilic warhead into a suitable position for covalent bond formation (Figure 2A). This step is reversible, as the molecule returns to the inactive configuration when the irradiation is stopped [11,13]. 

In contrast, photocaging is a concept where a bioactive compound is rendered unable to bind to its target by the attachment of a photocleavable protecting group (PPG) at a suitable position in the molecule [11,14,15]. If the photocleavable group is removed by irradiation, the binding affinity can be restored and, in the case of covalent inhibitors, the formation of the covalent bond to the target can take place (Figure 2B). This process is irreversible. 

**Figure 3:**
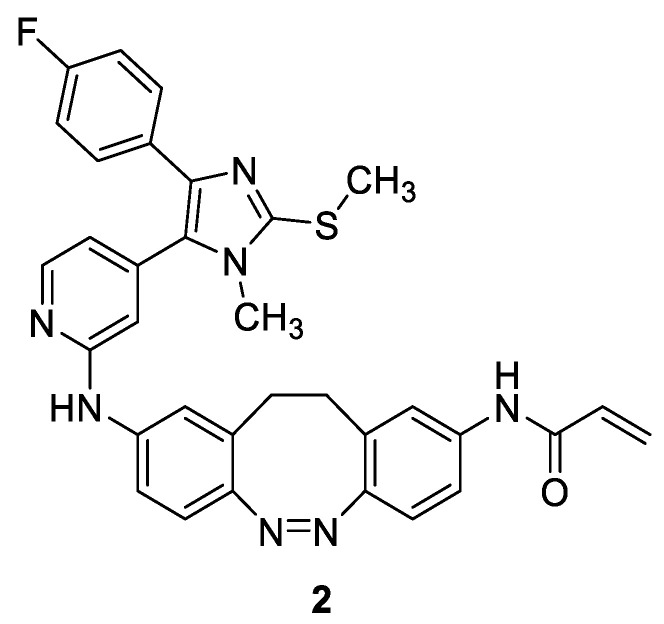
Structure of photoswitchable covalent JNK3 inhibitor **2**.

To date, photopharmacological approaches have been applied to several biologically relevant molecules including a variety of kinase inhibitors [16,17,18,19,20]. In collaboration with other research groups, some of us developed a diazocine-based photoswitchable JNK3 inhibitor **2** using compound **1** as the lead structure to acquire spatiotemporal control over the inhibition of JNK3 (Figure 3) [10]. The introduction of a diazocine photoswitch into the linker moiety of **1** allowed differentiation between an inactive (*cis*) and an active (*trans*) form, since only the *trans* configuration facilitates covalent engagement of Cys154. Light pulses at 390 nm proved sufficient to control the *trans*–*cis* switch, resulting in an increase in biochemical activity by a factor of 30. However, in the cellular NanoBRET^TM^ assay, this effect was less pronounced, as light pulses at 400 nm increased the inhibitory potency by a factor of only two [10].

**Figure 4:**
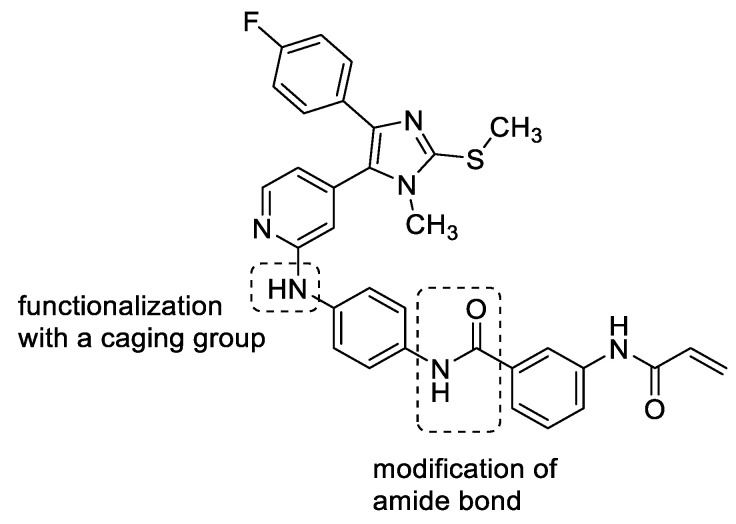
Aim of the present study. Inversion and methylation of the amide bond present between the two benzene rings. Installation of a photocleavable protecting group at the secondary amine at the pyridin-C2-amino function.

The aim of the present study was to design novel pyridinylimidazole-based covalent JNK3 inhibitors using compound **1** as the lead structure. First, the influence of the amide bond between the two benzene rings was investigated (Figure 4). Second, a photocleavable protecting group was introduced into the most promising modified covalent JNK3 inhibitor as a proof-of-concept study of photocaging this class of covalent JNK3 inhibitors. The hinge binding motif (pyridin-2-amino function) of **1** represents an essential structural feature for this inhibitor and its congeners to address the JNK3 ATP binding site (Figure 4). Consequently, we hypothesized this motif to be a suitable photocaging position, as the introduction of a bulky photocleavable protecting group is likely to impair the binding of the inhibitor to the hinge region of the targeted kinase, resulting in a weak or inactive compound.

## 2. Results and Discussion

### 2.1. Biological Evaluation

To evaluate the influence of the amide bond between the two benzene rings on the compound activity, we considered the inversion of this functionality as well as methylation of the nitrogen atom leading to compounds **8** and **13**, respectively (Table 1). Despite a two-fold decreased biological activity compared to parent compound **1** (IC_50_ = 6 nM), these derivatives remained potent JNK3 inhibitors with IC_50_ values in the low double-digit nanomolar range. Interestingly, replacement of the acrylamide warhead in **8** and **13** with an unreactive propionamide moiety (inhibitors **10** and **14**, respectively) was tolerated by the target enzyme. These data suggested that the strong biochemical potency of this compound series is predominantly achieved by reversible interactions of the scaffold to JNK3 with a minor contribution from the covalent bond formation. 

Next, the compound series was investigated in a NanoBRET^TM^ target engagement (TE) intracellular JNK3 assay. The data obtained from these experiments correlated well with the trends observed in the biochemical assay, as pyridinylimidazoles **8** and **13** displayed a 4-fold and 5.5-fold decreased binding affinity, respectively, compared to lead structure **1** (IC_50_ = 243 nM). Noteworthily, the propionamides **10** and **14** demonstrated substantially weaker IC_50_ values in the NanoBRET^TM^ assay than anticipated based on their biochemical potencies.

**Figure 5:**
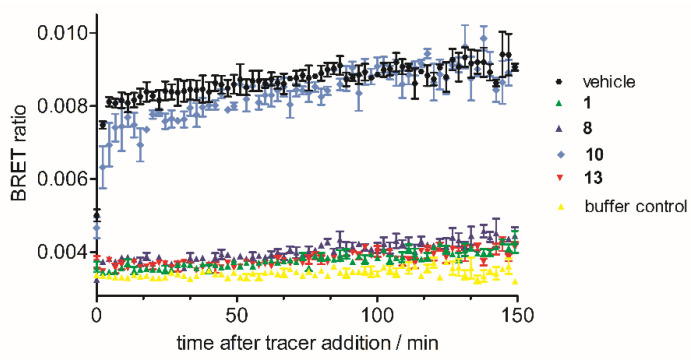
Kinetic analyses of compounds **1**, **8**, **10,** and **13**.

To study the putative covalent binding of inhibitors **8** and **13**, we next applied the compound washout method in the NanoBRET^TM^ assay [21]. Briefly, we established a protocol where the compound dissociation of **8** and **13** was determined indirectly by their ability to inhibit binding of the ATP-competitive tracer. The covalent inhibitor **1** was used as a positive control, and the propionamide **10** was used as a negative control. In this experiment, the saturated compound **10** was quickly displaced by the tracer, whereas inhibitors **8**, **13**, and lead compound **1** remained persistently bound to the target enzyme (Figure 5).

**Figure 6:**
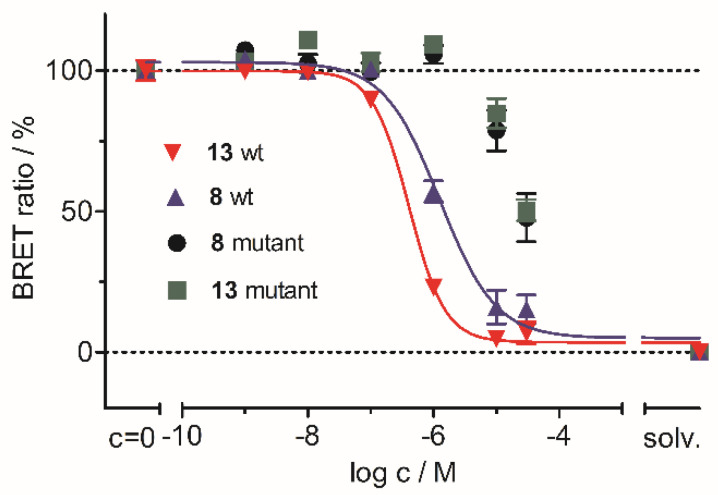
Determination of the IC_50_ values of **8** and **13** for wildtype JNK3-NLuc and for JNK3(C154A)-NLuc in a NanoBRET^TM^ TE intracellular JNK3 assay.

As these results indicated an irreversible binding mode of acrylamides **8** and **13** in the ATP binding site of JNK3, we further examined the covalent engagement of the targeted residue Cys154. To this end, we generated a JNK3(C154A)-NLuc mutant and determined the IC_50_ values of **8** and **13** for the modified enzyme. As expected, this single point mutation led to a dramatic decrease in the binding affinity of inhibitors **8** and **13** (Figure 6).

To assess the general reactivity of acrylamides **8** and **13** towards nucleophiles, we conducted a stability study in the presence of the physiological nucleophile glutathione (GSH) following a modified literature methodology (for details see Supplementary Materials) [22]. When exposed to a 200-fold excess of GSH at a physiological pH, acrylamides **8** and **13** demonstrated a similar degradation during 72 h compared to the chemical probe **JNK-IN-8.** These results indicate a favorable stability profile for the pyridinylimidazole-based covalent JNK3 inhibitors against nucleophiles (Figure S1, Supplementary Materials).

Based on our findings, we selected **13** as the most promising covalent JNK3 inhibitor of our novel series for the photocaging approach. We introduced a photocleavable 4,5-dimethoxy-2-nitrobenzyl protecting group on the pyridinyl amine nitrogen atom, resulting in compound **17** (Table 1), that, expectedly, led to a decrease in JNK3 binding by nearly one order of magnitude in the NanoBRET^TM^ assay.

**Figure 7:**
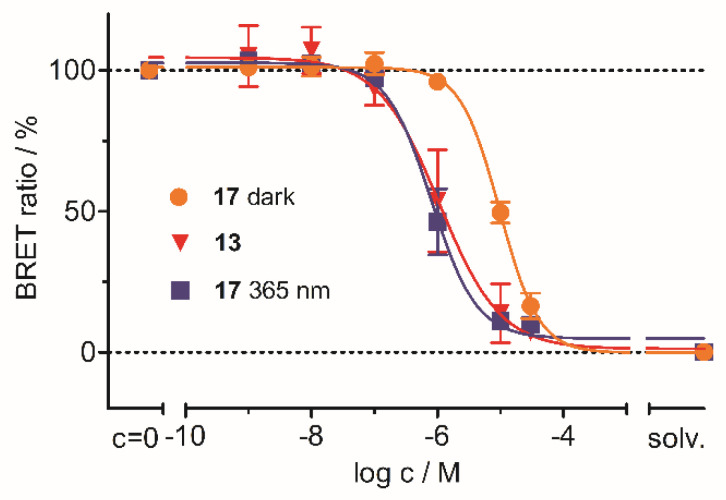
Photocleavage experiments of **17** in the cellular assay setting. Here, **17** 365 nm refers to the irradiated sample; **17** dark refers to the data collected without irradiation. Inhibitor **13** (uncaged analog of **17**) served as the reference compound.

Finally, this assay was repeated with compound **17**, applying UV irradiation to achieve quantitative cleavage of the PPG. The selected conditions (light with a wavelength of 365 nm for 8 min) afforded full recovery of the binding affinity, as **17** and its unprotected parent compound **13** exhibited extremely similar IC_50_ values in this experiment (Figure 7). These results confirmed the successful application of the photocaging strategy to our covalent JNK3 inhibitor **13**. 

### 2.2. Chemistry

**Scheme 1:**
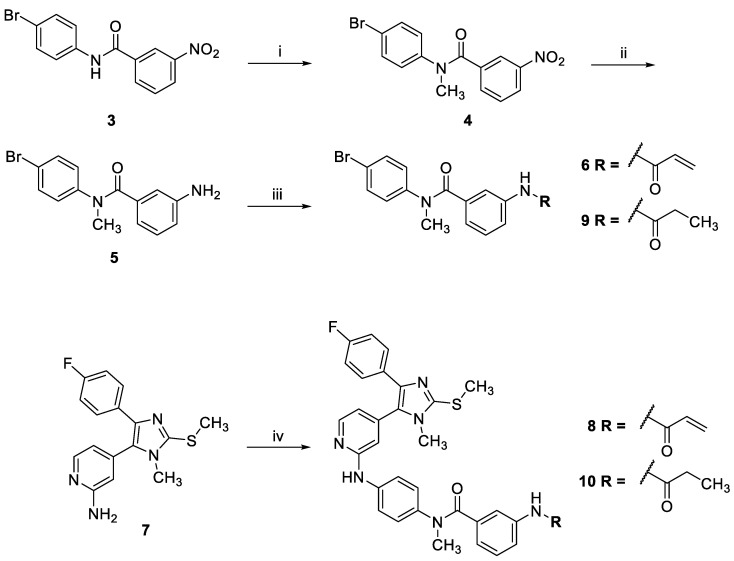
Synthesis of *N*-methyl derivatives **8** and **10**. (i) Methyl iodide, NaH, THF, Ar, r.t., 6 h, 88%; (ii) Sn(II)Cl_2_, EtOH, reflux, 16 h, 58%; (iii) acryloyl chloride in the case of **6** or propionic anhydride in the case of **9**, DIPEA, 1,4-dioxane, Ar, r.t., 2–3 h, 44%; (iv) **6** in the case of the synthesis of **8** or **9** in the case of the synthesis of **10**, BrettPhos Pd precatalyst, Cs_2_CO_3_, 1,4-dioxane/^t^BuOH, Ar, 115 °C, 16 h, 9–20%.

Pyridinylimidazole **8** was prepared using a similar synthetic strategy as reported for lead structure **1** (Scheme 1) [9]. First, the side chain **6** bearing the electrophilic warhead was prepared starting from *N*-(4-bromophenyl)-3-nitrobenzamide (**3**) in three steps. The amide function present in **3** was methylated using methyl iodide as the alkylating agent. Then, the nitro group was reduced by treatment with tin(II) chloride followed by introduction of the acrylamide warhead using acryloyl chloride. Finally, side chain **6** was coupled with the reported 4-(4-(4-fluorophenyl)-1-methyl-2-(methylthio)-1*H*-imidazol-5-yl)pyridin-2-amine (**7**) [23,24] under Buchwald-Hartwig aryl amination conditions to yield covalent inhibitor **8** (for a synthetic pathway toward compound **7**, see Scheme S1). For the preparation of the saturated counterpart of **8** (compound **10**), aniline derivative **5** was reacted with propionic anhydride instead to yield the saturated side chain **9**, which was coupled with pyridinyl imidazole **7** to obtain **10** (Scheme 1).

**Scheme 2:**
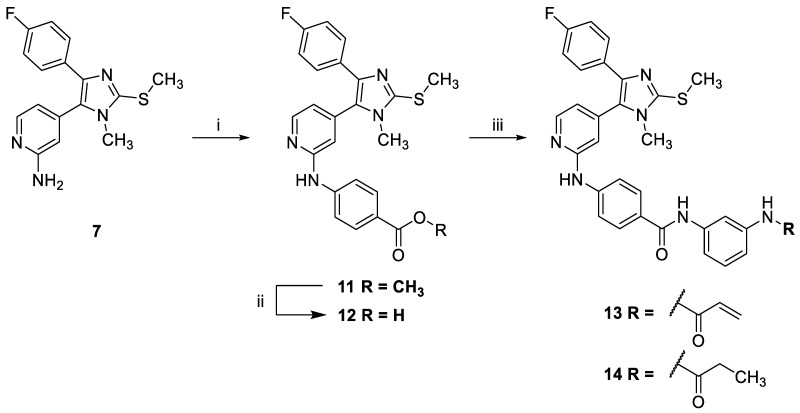
Synthesis of inverse amide derivatives **13** and **14**. (i) Methyl 4-bromobenzoate, BrettPhos Pd precatalyst, Cs_2_CO_3_, 1,4-dioxane/^t^BuOH, Ar, 115 °C, 16 h, 73%; (ii) LiOH in THF/MeOH/H_2_O, r.t., 37 h, 95%; (iii) **12**, *N*-(3-aminophenyl)acrylamide in the case of the synthesis of **13** or *N*-(3-aminophenyl)propionamide in the case of the synthesis of **14**, HATU, DIPEA, DCM, r.t., 22–27 h, 43–52%.

Pyridinylimidazole **13** and its saturated counterpart **14** were synthesized in three steps starting from compound **7** (Scheme 2). First, **7** was reacted with methyl 4-bromobenzoate under Buchwald-Hartwig conditions to yield compound **11**. Then, the ester function present in **11** was converted into the free carboxylic acid **12** under basic conditions [25]. Finally, the carboxylic acid **12** was coupled with commercially available *N*-(3-aminophenyl)acrylamide or *N*-(3-aminophenyl)propionamide, respectively, using *O*-(7-azabenzotriazol-1-yl)-*N*,*N*,*N′*,*N′*-tetramethyluronium-hexafluorphosphate (HATU) as a coupling reagent to yield the covalent inhibitor **13** and its saturated counterpart **14**, respectively.

**Scheme 3:**
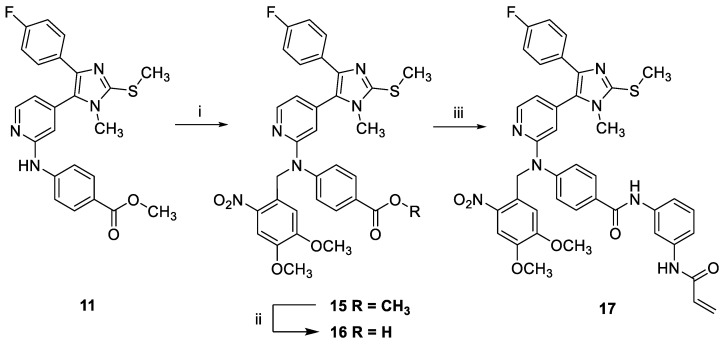
Synthesis of photocaged compound **17**. (i) 4,5-Dimethoxy-2-nitrobenzyl bromide, NaH, DMF, Ar, −10 °C to r.t., 16 h, 64%; (ii) LiOH in THF/MeOH/H_2_O, r.t., 17 h, 87%; (iii) **16**, *N*-(3-aminophenyl)acrylamide, HATU, DIPEA, DCM, r.t., 46 h, 21%.

The synthesis of the photocaged compound **17** started from intermediate **11** (Scheme 3). First, the PPG was installed under nucleophilic substitution conditions using sodium hydride as base and 4,5-dimethoxy-2-nitrobenzyl bromide as alkylating reagent according to a modified literature protocol [26]. In the next step, the ester function of **15** was saponified, providing carboxylic acid **16**. Finally, the electrophilic warhead was introduced by amide coupling of **16** with *N*-(3-aminophenyl)acrylamide to obtain pyridinylimidazole **17**.

### 2.3. Conclusions

Starting from the potent covalent inhibitor acrylamido-*N*-(4-((4-(4-(4-fluorophenyl)-1-methyl-2-(methylthio)-1*H*-imidazol-5-yl)pyridin-2-yl)amino)phenyl)benzamide (**1**), we herein designed novel pyridinylimidazole-based inhibitors of JNK3 by modifying the amide bond moiety in the linker. The *N*-methylation, as well as the inversion of the amide bond, were both tolerated by the target enzyme, leading to inhibitors with IC_50_ values in the low double-digit nanomolar range in a radiometric JNK3 assay. The compounds were further evaluated in a NanoBRET^TM^ JNK3 assay to investigate the target engagement in a cellular setting. Here, acrylamides **8** and **13** exhibited persistent binding to JNK3 in compound washout experiments, along with a loss in binding affinity for a newly generated JNK3(C154A)-NLuc mutant, confirming covalent bond formation to the targeted residue Cys154. Moreover, these inhibitors displayed a favorable stability against nucleophiles in a glutathione reactivity assay. The most promising candidate of the series, compound **13**, was selected for the photocaging approach and equipped with a 4,5-dimethoxy-2-nitrobenzyl protecting group to impair its binding to the hinge region of JNK3. The introduction of this PPG in **17** dramatically decreased its intracellular JNK3 binding affinity, which was fully restored by UV irradiation at a wavelength of 365 nm within 8 min. Our results demonstrate that the concept of photocaging can be exploited to control kinase inhibitor binding affinity by nearly one order of magnitude in live cells. 

## 3. Materials and Methods

### 3.1. Chemistry

#### 3.1.1. General Information

All reagents and solvents were of commercial quality and were utilized without further purification. High-performance liquid chromatography (HPLC) was used to determine the retention times of all reported compounds, as well as the purity of the test compounds, which was >95%, if not stated otherwise under Section 3.1.3. The chromatographic separation was carried out on an Agilent 1100 Series HPLC system from Agilent Technologies (Santa Clara, CA, USA), equipped with an ultraviolet diode array detector with detecting at 254 nm and an XBridge^TM^ C18 column (150 × 4.6 mm, 5 µm) from Waters (Milford, MA, USA). The oven temperature was set to 30 °C, the injection volume was 5 μL, and the flow was 1.5 mL/min. Compounds were eluted using the following gradient: mobile phase A—0.01 M KH_2_PO_4_ (pH 2.3); mobile phase B—MeOH; 40% B to 85% B in 8 min; 85% B for 5 min; 85% B to 40% B in 2 min; 40% B for 1 min. Purifications by preparative HPLC were carried out with a system from Knauer (Berlin, Germany). The system consisted of two Knauer K-1800 pumps, a Knauer K-2001 detector, and a Gemini^®^ 5 µm NX-C18 110 Å (250 × 21.2 mm) LC column from Phenomenex (Torrance, CA, USA). Compounds were eluted using the gradient as follows. Mobile phase C—0.1% aqueous TFA; mobile phase B—MeOH; 40% B to 90% B in 20 min; 90% B for 5 min. Compounds which were finally purified by preparative HPLC were obtained as hydrotrifluoroacetate salts as confirmed by ^19^F NMR analysis. Column chromatography was performed on an Interchim PuriFlash XS520Plus automated flash chromatography system using Geduran Si_60_ 40–63 µm silica from Merck (Darmstadt, Germany) or commercial 50 µm silica columns from Interchim (Montluçon, France). The NMR spectra were measured on an Avance 300 (300 MHz for ^1^H, 75 MHz for ^13^C) or an Avance 400 (400 MHz for ^1^H, 100 MHz for ^13^C, 377 MHz for ^19^F) NMR spectrometer from Bruker (Billerica, BA, USA). Chemical shifts are reported in parts per million (ppm) relative to tetramethylsilane. All spectra were calibrated against the (residual proton) peak of the deuterated solvent. Mass spectrometry was carried out on a Finnigan MAT 95, a Thermo Quest Finnigan TSQ 7000, a Finnigan MATSSQ 710 A, or an Agilent 6540 UHD Accurate-Mass Q-TOF liquid chromatography coupled electrospray ionization mass spectrometer (LC-ESI-MS) from Agilent Technologies (Santa Clara, CA, USA) at the analytical department of the University of Regensburg. 

#### 3.1.2. General Procedures

(1) General Procedure A (Buchwald-Hartwig Arylamination)

4-(4-(4-Fluorophenyl)-1-methyl-2-(methylthio)-1*H*-imidazol-5-yl)pyridin-2-amine (**7**) [23,24] (1.0 equiv.), the respective aryl bromide (1.1 equiv.), Cs_2_CO_3_ (2.5 equiv.), and BrettPhos Pd precatalyst (5 mol%) were added to a crimp top vial. A mixture of dry 1,4-dioxane and ^t^BuOH (4:1) was added to the solids, and the vial was closed with a crimp cap equipped with a septum. The reaction mixture was bubbled with argon for 20 to 25 min and subsequently stirred for 16 h at 115 °C under an argon atmosphere. The reaction was quenched with a 1:1 mixture of saturated NH_4_Cl solution and water. The mixture was extracted with EtOAc (3x). The combined organic phases were dried over MgSO_4_, filtered, and the solvents were removed under vacuum. The product was obtained after purification via flash chromatography, preparative HPLC, or recrystallization.

(2) General Procedure B (HATU-mediated Amide Coupling)

The respective carboxylic acid (1.0 equiv.) and HATU (1.1 equiv.) were stirred in dry DCM (90 mL/mmol) at room temperature under an argon atmosphere. After 30 min, a solution of the appropriate aniline (1.1 equiv.) and DIPEA (3.0 equiv.) in 1–2 mL of dry DCM was added to the mixture. The solution was stirred at room temperature for 22–27 h. The reaction was quenched by the addition of saturated NaCl solution and extracted with EtOAc (3x). The combined organic phases were dried over MgSO_4_, filtered, and the solvents were removed under vacuum. The product was obtained after purification via flash chromatography, preparative HPLC, or recrystallization. 

#### 3.1.3. Detailed Procedures

*N*-(4-Bromophenyl)-*N*-methyl-3-nitrobenzamide (**4**)

*N*-(4-Bromophenyl)-3-nitrobenzamide (**3**) (1.8 g, 5.76 mmol) [9] was dissolved in dry THF (50 mL) under an argon atmosphere, and the solution was cooled in an ice bath. Sodium hydride (461.0 mg of a 60% suspension on mineral oil, 11.5 mmol) was added in portions. After stirring at room temperature for 30 min the mixture was cooled with a MeOH-ice bath to −10 °C. Methyl iodide (0.9 mL, 14.4 mmol) was added, and the solution was left to warm to room temperature and stirred for 6 h, when full conversion was confirmed by TLC. All volatiles were removed under vacuum, and saturated NaCl solution as well as EtOAc were added to the residue. Phases were separated, and the aqueous phase was extracted with EtOAc (3x). The combined organic phases were dried over MgSO_4_, filtered, and the solvents were removed under vacuum. The brown oily residue was treated with a 3:1 mixture of PE and EtOAc with mild warming resulting in the formation of a precipitate. The supernatant was carefully removed, and the solid residue was dried under vacuum. The product was obtained as 1.7 g (88%) of a dark yellow solid. ^1^H NMR (300 MHz, DMSO-*d_6_*) δ 8.15 (m, 2H), 7.66 (m, 1H), 7.56 (m, 1H), 7.48 (m, 2H), 7.24 (m, 2H), 3.38 (s, 3H); ESI-MS (m/z)— 335.0, 337.0 [M+H]^+^, representing the two bromine isotopologues; HPLC *t_R_* = 6.82 min.

3-Amino-*N*-(4-bromophenyl)-*N*-methylbenzamide (**5**)

Compound **4** (1.7 g, 5.07 mmol) and Sn(II)Cl_2_·2H_2_O (5.7 g, 25.35 mmol) were dissolved in EtOH (50 mL). The solution was stirred at reflux temperature for 16 h. After cooling down, the solvent was removed under vacuum. The residue was carefully mixed with saturated NaHCO_3_ solution and EtOAc and then filtered. The phases of the filtrate were separated, and the aqueous phase was extracted with EtOAc (3x). The combined organic phases were dried over MgSO_4_, filtered, and the solvents were removed under vacuum. The crude product (900 mg; 58%) was used without further purification. ^1^H NMR (300 MHz, DMSO-*d_6_*) δ 7.45 (m, 2H), 7.10 (m, 2H), 6.83 (t, *J* = 7.8, 1H), 6.50 (m, 2H), 6.27 (m, 1H), 5.16 (s, 2H), 3.31 (s, 3H); ^13^C NMR (101 MHz, DMSO-*d_6_*) δ 169.8, 144.0, 136.8, 131.9, 131.3, 128.8, 128.4, 118.8, 116.7, 115.2, 113.6, 37.7; HR-ESI-MS (m/z)—calculated 305.0284 [M+H]^+^, found 305.0286 [M+H]^+^; HPLC *t_R_* = 4.86 min.

3-Acrylamido-*N*-(4-bromophenyl)-*N*-methylbenzamide (**6**)

Compound **5** (1.0 g; 3.28 mmol) and DIPEA (1.1 g, 8.19 mmol) were stirred in dry 1,4-dioxane (20 mL) under an argon atmosphere. The stirring mixture was cooled to 0 °C with an ice bath, and acryloyl chloride (326.2 mg, 3.60 mmol) was added. The solution was allowed to warm up to room temperature and stirred for 2 h when full conversion was confirmed via HPLC. The reaction was quenched by the addition of saturated NH_4_Cl solution and extracted with EtOAc (3x). The combined organic phases were dried over MgSO_4_, filtered, and the solvents were removed under vacuum. The crude product (520 mg; 44%) was used in the next step without further purification. A small portion was purified via flash chromatography (SiO_2_, PE:EtOAc:MeOH 50:47.5:2.5) for analytical purposes. ^1^H NMR (300 MHz, DMSO-*d_6_*) δ 10.19 (s, 1H), 7.79 (s, 1H), 7.61–7.54 (m, 1H), 7.49–7.40 (m, 2H), 7.21–7.08 (m, 3H), 6.85 (d, *J* = 7.7 Hz, 1H), 6.41 (dd, *J* = 17.0, 9.9 Hz, 1H), 6.25 (dd, *J* = 17.0, 2.2 Hz, 1H), 5.75 (dd, *J* = 9.9, 2.2 Hz, 1H), 3.35 (s, 3H); ^13^C NMR (75 MHz, DMSO-*d_6_*) δ 169.2, 163.2, 143.8, 138.8, 136.6, 132.0, 131.7, 129.0, 128.2, 127.2, 123.1, 120.3, 119.1, 119.0, 37.8; HR-ESI-MS (m/z)—calculated 359.0390 [M+H]^+^, found 359.0392 [M+H]^+^; HPLC *t_R_* = 6.33 min.

3-Acrylamido-*N*-(4-((4-(4-(4-fluorophenyl)-1-methyl-2-(methylthio)-1*H*-imidazol-5-yl)pyridin-2-yl)amino)phenyl)-*N*-methylbenzamide hydrotrifluoroacetate (**8**)

Compound **8** was synthesized according to general procedure A from compound **7** (80.0 mg, 0.25 mmol), compound **21** (102.0 mg, 0.28 mmol), BrettPhos Pd G3 (5.8 mg, 0.01 mmol), and Cs_2_CO_3_ (207.0 mg, 0.63 mmol). The crude material was purified via flash chromatography (DCM:MeOH gradient elution from 99:1 to 95:5) and subsequently via preparative HPLC. The product was obtained as 31 mg (20%) of a yellow solid. ^1^H NMR (400 MHz, DMSO-*d_6_*) δ 10.14 (s, 1H), 9.36 (s, 1H), 8.21 (d, *J* = 5.1 Hz, 1H), 7.75 (s, 1H), 7.56–7.35 (m, 5H), 7.19–6.97 (m, 5H), 6.89–6.69 (m, 3H), 6.38 (dd, *J* = 16.9, 10.1 Hz, 1H), 6.22 (d, *J* = 16.5 Hz, 1H), 5.73 (d, *J* = 10.2 Hz, 1H), 3.43 (s, 3H), 3.33 (s, 3H), 2.66 (s, 3H); ^13^C NMR (101 MHz, DMSO-*d_6_*) δ 169.3, 163.2, 161.3 (d, *J* = 244.2 Hz), 155.6, 147.2, 144.0, 139.09, 139.05, 138.6, 137.6, 137.2, 136.3, 131.7, 129.5 (d, *J* = 3.1 Hz), 128.7 (d, *J* = 8.0 Hz), 128.1, 128.0, 127.4, 127.1, 123.1, 119.9, 119.1, 118.8, 115.4 (d, *J* = 21.4 Hz), 111.8, 38.0, 31.9, 15.5; ^19^F NMR (377 MHz, DMSO-*d_6_*) δ -74.3, -114.5; HR-ESI-MS (m/z)—calculated 593.2129 [M+H]^+^, found 593.2136 [M+H]^+^; HPLC *t_R_* = 6.42 min.

Synthesis of *N*-(4-Bromophenyl)-*N*-methyl-3-propionamidobenzamide (**9**)

Compound **6** (450.0 mg, 1.47 mmol) and DIPEA (476.5 mg, 3.69 mmol) were stirred in dry 1,4-dioxane (15 mL) under an argon atmosphere. The mixture was cooled to 0 °C with an ice bath and propionic anhydride (230.3 mg, 1.77 mmol) was added. The solution was allowed to warm up to room temperature and was stirred for 3 h when full conversion was confirmed via HPLC. The reaction was quenched by the addition of saturated NH_4_Cl solution and extracted with EtOAc (3x). The combined organic phases were dried over MgSO_4_, filtered, and the solvents were removed under vacuum. The crude product was obtained as 280 mg of a brown oil (53%) and used in the next step without further purification. A small portion was purified via flash chromatography (SiO_2_, DCM:MeOH 97:3) for analytical purposes. ^1^H NMR (300 MHz, DMSO-*d_6_*) δ 9.90 (s, 1H), 7.69 (s, 1H), 7.54–7.40 (m, 3H), 7.20–7.05 (m, 3H), 6.79 (d, *J* = 7.6 Hz, 1H), 3.34 (s, 3H), 2.29 (q, *J* = 7.5 Hz, 2H), 1.05 (t, *J* = 7.5 Hz, 3H); ^13^C NMR (75 MHz, DMSO-*d_6_*) δ 172.1, 169.3, 143.8, 139.2, 136.5, 131.9, 129.0, 128.1, 122.5, 119.9, 119.0, 118.7, 37.8, 29.5, 9.6; HR-ESI-MS (m/z)—calculated 361.0546 [M+H]^+^, found 361.0549 [M+H]^+^; HPLC *t_R_* = 6.28 min.

*N*-(4-((4-(4-(4-Fluorophenyl)-1-methyl-2-(methylthio)-1*H*-imidazol-5-yl)pyridin-2-yl)amino)phenyl)-*N*-methyl-3-propionamidobenzamide (**10**)

Compound **10** was synthesized according to general procedure A from compound **7** (200.0 mg, 0.64 mmol), **9** (253.0 mg, 0.70 mmol), BrettPhos Pd G3 (14.4 mg, 0.03 mmol), and Cs_2_CO_3_ (518.0 mg, 1.60 mmol) in a 4:1 mixture of dry 1,4-dioxane and ^t^BuOH (15 mL). The crude material was purified via flash chromatography (DCM:MeOH gradient elution from 98:2 to 95:5). The product was obtained as 33 mg (9%) of an off-white solid. ^1^H NMR (400 MHz, DMSO-*d_6_*) δ 9.85 (s, 1H), 9.18 (s, 1H), 8.23 (d, *J* = 5.2 Hz, 1H), 7.68 (s, 1H), 7.54 (d, *J* = 8.8 Hz, 2H), 7.48–7.38 (m, 3H), 7.15–6.96 (m, 5H), 6.81–6.72 (m, 2H), 6.69 (s, 1H), 3.40 (s, 3H), 3.31 (s, 3H), 2.63 (s, 3H), 2.27 (q, *J* = 7.5 Hz, 2H), 1.04 (t, *J* = 7.5 Hz, 3H); ^13^C NMR (101 MHz, DMSO-*d_6_*) δ 172.0, 169.5, 161.1 (d, *J* = 243.5 Hz), 156.1, 148.2, 143.7, 139.6, 139.0, 139.0, 137.1, 136.8, 130.5 (d, *J* = 2.9 Hz), 128.3 (d, *J* = 8.1 Hz), 128.1, 127.8, 127.3, 122.5, 119.5, 118.7, 118.2, 115.4, 115.2 (d, *J* = 21.5 Hz), 111.7, 38.0, 31.6, 29.5, 15.3, 9.6; HR-ESI-MS (m/z)—calculated 595.2286 [M+H]^+^, found 595.2293 [M+H]^+^; HPLC *t_R_* = 7.21 min.

Methyl 4-((4-(4-(4-fluorophenyl)-1-methyl-2-(methylthio)-1*H*-imidazol-5-yl)pyridin-2-yl)amino)benzoate (**11**)

Compound **11** was synthesized according to general procedure A from compound **7** (250.0 mg, 0.79 mmol), methyl 4-bromobenzoate (257.0 mg, 1.19 mmol), BrettPhos Pd G4 (11.0 mg, 0.01 mmol), and Cs_2_CO_3_ (648.0 mg, 1.98 mmol) in a 4:1 mixture of dry 1,4-dioxane and ^t^BuOH (20 mL). The crude material obtained by extraction was treated with MeOH resulting in a precipitate. The yellow supernatant was carefully removed, and the remaining solid was dried under vacuum. The product was obtained as 260 mg (73%) of a pale yellow solid. ^1^H NMR (300 MHz, DMSO-*d_6_*) δ 9.65 (s, 1H), 8.36 (d, *J* = 5.2 Hz, 1H), 7.90–7.78 (m, 5H), 7.49–7.41 (m, 2H), 7.18–7.09 (m, 2H), 6.90 (dd, *J* = 5.2, 1.4 Hz, 1H), 6.86–6.83 (m, 1H), 3.80 (s, 3H), 3.44 (s, 3H), 2.66 (s, 3H); ^13^C NMR (75 MHz, DMSO-*d_6_*) δ 166.1, 161.1 (d, *J* = 243.5 Hz), 155.7, 148.3, 146.0, 143.9, 139.3, 137.0, 130.5 (d, *J* = 3.0 Hz), 130.4, 128.4 (d, *J* = 8.1 Hz), 128.0, 120.9, 116.8, 116.5, 115.3 (d, *J* = 21.5 Hz), 112.6, 51.7, 31.6, 15.3; HR-ESI-MS (m/z)—calculated 449.1442 [M+H]^+^, found 449.1447 [M+H]^+^; HPLC *t_R_* = 9.14 min.

4-((4-(4-(4-Fluorophenyl)-1-methyl-2-(methylthio)-1*H*-imidazol-5-yl)pyridin-2-yl)amino)benzoic acid (**12**)

Compound **11** (110.0 mg, 0.25 mmol) was dissolved in a mixture of THF, MeOH, and 1.5 M aqueous LiOH solution (6:3:1) (9 mL). The mixture was stirred at room temperature for 40 h, when incomplete conversion was observed via HPLC. Additional 1.5 M aqueous LiOH solution (1 mL) was added, and then stirring continued at room temperature overnight. The solvents were removed under vacuum, and the residue was dissolved in water and acidified with acetic acid until a precipitate formed. The mixture was stored in the fridge for 2 h and then filtered. The filter cake was washed with demineralized water and dried overnight. The product was obtained as 102 mg (95%) of a yellow solid. ^1^H NMR (300 MHz, DMSO-*d_6_*) δ 12.54 (br s, 1H), 9.60 (s, 1H), 8.35 (d, *J* = 4.8 Hz, 1H), 7.95–7.73 (m, 4H), 7.52–7.40 (m, 2H), 7.21–7.05 (m, 2H), 6.93–6.80 (m, 2H), 3.43 (s, 3H), 2.65 (s, 3H); ^13^C NMR (75 MHz, DMSO-*d_6_*) δ 167.3, 161.1 (d, *J* = 243.6 Hz), 155.8, 148.4, 145.6, 143.9, 139.3, 137.1, 130.6, 128.4 (d, *J* = 7.9 Hz), 128.0, 122.2, 116.8 (signal overlap assumed), 116.3, 115.3 (d, *J* = 21.5 Hz), 112.5, 31.6, 15.3; HR-ESI-MS (m/z)—calculated 435.1286 [M+H]^+^, found 435.1290 [M+H]^+^; HPLC *t_R_* = 7.40 min.

*N*-(3-Acrylamidophenyl)-4-((4-(4-(4-fluorophenyl)-1-methyl-2-(methylthio)-1*H*-imidazol-5-yl)pyridin-2-yl)amino)benzamide hydrotrifluoroacetate (**13**)

Compound **13** was synthesized according to general procedure B from carboxylic acid **12** (40.0 mg, 0.09 mmol), *N*-(3-aminophenyl)acrylamide (17.0 mg, 0.10 mmol), HATU (38.5 mg, 0.10 mmol), and DIPEA (35.0 mg, 0.27 mmol) in dry DCM (9 mL). The reaction was stopped after 27 h, when full conversion was confirmed by TLC. The crude material obtained by extraction was purified via preparative HPLC. The product was obtained as 28 mg (52%) of a yellow solid. ^1^H NMR (400 MHz, DMSO-*d_6_*) δ 10.17 (s, 1H), 10.08 (s, 1H), 9.63 (s, 1H), 8.35 (d, *J* = 4.8 Hz, 1H), 8.19 (s, 1H), 7.92 (d, *J* = 8.4 Hz, 2H), 7.79 (d, *J* = 8.4 Hz, 2H), 7.53–7.39 (m, 4H), 7.32–7.11 (m, 3H), 6.92–6.84 (m, 2H), 6.48 (dd, *J* = 16.9, 10.1 Hz, 1H), 6.26 (d, *J* = 16.8 Hz, 1H), 5.75 (d, *J* = 10.1 Hz, 1H), 3.48 (s, 3H), 2.68 (s, 3H); ^13^C NMR (101 MHz, DMSO-*d_6_*) δ 165.0, 163.1, 161.3 (d, *J* = 244.3 Hz), 148.0, 144.3, 143.9, 139.7, 139.2, 138.9, 136.2, 132.0, 128.7 (m), 128.1, 126.7, 126.5, 117.0, 116.0, 115.7, 115.4 (d, *J* = 21.6 Hz), 114.6, 112.3, 111.6, 31.9, 15.5; ^19^F NMR (377 MHz, DMSO-*d_6_*) δ -74.3, -114.4; exact mass: 578.2, HR-ESI-MS (m/z) —calculated 579.1973 [M+H]^+^, found 579.1980 [M+H]^+^; HPLC *t_R_* = 8.12 min.

4-((4-(4-(4-Fluorophenyl)-1-methyl-2-(methylthio)-1*H*-imidazol-5-yl)pyridin-2-yl)amino)-*N*-(3-propionamidophenyl)benzamide (**14**)

Compound **14** was synthesized according to general procedure B from intermediate **12** (39.0 mg, 0.09 mmol), *N*-(3-aminophenyl)propionamide (16.1 mg, 0.10 mmol), HATU (37.3 mg, 0.10 mmol), and DIPEA (35.0 mg, 0.27 mmol) in dry DCM (6 mL). The reaction was stopped after 22 h, when complete conversion was confirmed by HPLC. The crude material obtained from extraction was treated with a mixture of MeOH:H_2_O (1:1) resulting in a precipitate, which was filtered off, dried, and purified via flash chromatography (SiO_2_, PE:(EtOAc:MeOH 95:5)–2:3). The product was obtained as 22 mg (43%) of a pale yellow solid. ^1^H NMR (400 MHz, DMSO-*d_6_*) δ 10.02 (s, 1H), 9.87 (s, 1H), 9.54 (s, 1H), 8.35 (d, *J* = 5.2 Hz, 1H), 8.10 (s, 1H), 7.92 (d, *J* = 8.8 Hz, 2H), 7.81 (d, *J* = 8.8 Hz, 2H), 7.50–7.43 (m, 2H), 7.42–7.38 (m, 1H), 7.36–7.32 (m, 1H), 7.23 (t, *J* = 8.1 Hz, 1H), 7.18–7.11 (m, 2H), 6.89–6.82 (m, 2H), 3.45 (s, 3H), 2.66 (s, 3H), 2.33 (q, *J* = 7.5 Hz, 2H), 1.09 (t, *J* = 7.5 Hz, 3H); ^13^C NMR (101 MHz, DMSO-*d_6_*) δ 171.9, 165.0, 161.1 (d, *J* = 243.7 Hz), 155.9, 148.3, 144.5, 143.8, 139.7, 139.5, 139.2, 136.9, 130.4 (d, *J* = 2.5 Hz), 128.7, 128.5, 128.4 (d, *J* = 8.0 Hz), 128.0, 126.3, 116.8, 116.1, 115.3 (d, *J* = 21.4 Hz), 115.2, 114.3, 112.2, 111.4, 31.7, 29.5, 15.3, 9.7; HR-ESI-MS (m/z)—calculated 581.2129 [M+H]^+^, found 581.2136 [M+H]^+^; HPLC *t_R_* = 8.07 min.

Methyl 4-((4,5-dimethoxy-2-nitrobenzyl)(4-(4-(4-fluorophenyl)-1-methyl-2-(methylthio)-1*H*-imidazol-5-yl)pyridine-2-yl)amino)benzoate (**15**)

Here, NaH (19.2 mg of a 60% dispersion on mineral oil, 0.48 mmol) was added in portions to an ice-cooled stirring solution of compound **11** (143.0 mg, 0.32 mmol) in dry DMF (3.5 mL). The mixture was allowed to warm up to room temperature and was stirred for 30 min. The solution was cooled to −10 °C with a MeOH-ice bath. Subsequently, 6-nitroveratryl bromide (96.6 mg, 0.38 mmol), dissolved in 0.5 mL of dry DMF, was added. The mixture was stirred at −10 °C for 2.5 h when full conversion was confirmed via HPLC. The reaction was stopped by the addition of saturated NaHCO_3_ solution and the mixture was extracted with EtOAc (3x). The combined organic phases were dried over MgSO_4_, filtered, and the solvents were removed under vacuum. The crude material was purified via flash chromatography (PE:EtOAc gradient elution from 7:3 to 4:6). The product was obtained as 139 mg (64%) of a yellow oil. ^1^H NMR (300 MHz, CDCl_3_) δ 8.30 (dd, *J* = 4.8, 1.1 Hz, 1H), 7.88–7.82 (m, 2H), 7.72 (s, 1H), 7.40–7.31 (m, 2H), 7.15–7.07 (m, 2H), 7.00–6.88 (m, 3H), 6.78–6.73 (m, 2H), 5.67 (s, 2H), 3.94 (s, 3H), 3.89 (s, 3H), 3.68 (s, 3H), 3.44 (s, 3H), 2.67 (s, 3H); ESI-MS (m/z)—644.2 [M+H]^+^; HPLC *t_R_* = 10.02 min.

4-((4,5-Dimethoxy-2-nitrobenzyl)(4-(4-(4-fluorophenyl)-1-methyl-2-(methylthio)-1*H*-imidazol-5-yl)pyridin-2-yl)amino)benzoic acid (**16**)

Compound **15** (139.0 mg, 0.22 mmol) was dissolved in a mixture of THF, MeOH, and 1.5 M aqueous LiOH solution (6:3:1) (30 mL). The reaction mixture was stirred at room temperature for 17 h and then stopped by the addition of water. The pH was adjusted to 2-3 using concentrated HCl, and the aqueous phase was extracted with EtOAc (3x). The combined organic phases were dried over MgSO_4_, filtered, and the solvents were removed under vacuum. The crude product (118 mg) was used in the next step without further purification. HPLC *t_R_* = 9.108 min.

*N*-(3-Acrylamidophenyl)-4-((4,5-dimethoxy-2-nitrobenzyl)(4-(4-(4-fluorophenyl)-1-methyl-2-(methylthio)-1*H*-imidazol-5-yl)pyridin-2-yl)amino)benzamide hydrotrifluoroacetate (**17**)

Compound **17** was synthesized according to general procedure B from crude carboxylic acid **16** (87.0 mg), *N*-(3-aminophenyl)acrylamide (25.0 mg, 0.15 mmol), HATU (58.0 mg, 0.15 mmol), and DIPEA (54.3 mg, 0.42 mmol) in dry DCM (15 mL). The reaction mixture was stirred at room temperature for 46 h. The crude material obtained from extraction was purified via flash chromatography (PE:EtOAc gradient elution from 1:1 to 1:3), followed by a second purification step via preparative HPLC. The product was obtained as 22 mg (21%) of an orange-brown solid. ^1^H NMR (400 MHz, DMSO-*d_6_*) δ 10.20–10.14 (m, 2H), 8.31 (d, *J* = 5.1 Hz, 1H), 8.19–8.15 (m, 1H), 7.84 (d, *J* = 8.7 Hz, 2H), 7.69 (s, 1H), 7.48–7.44 (m, 1H), 7.41–7.34 (m, 5H), 7.26 (t, *J* = 8.1 Hz, 1H), 7.18–7.11 (m, 2H), 6.93 (s, 1H), 6.91 (dd, *J* = 5.2, 1.2 Hz, 1H), 6.82 (s, 1H), 6.47 (dd, *J* = 17.0, 10.1 Hz, 1H), 6.26 (dd, *J* = 17.0, 2.0 Hz, 1H), 5.75 (dd, *J* = 10.1, 2.0 Hz, 1H), 5.61 (s, 2H), 3.84 (s, 3H), 3.64 (s, 3H), 3.41 (s, 3H), 2.62 (s, 3H); ^13^C NMR (101 MHz, DMSO-*d_6_*) δ 164.7, 163.1, 161.3 (d, *J* = 244.3 Hz), 157.4, 153.1, 147.3, 147.2, 143.8, 140.1, 139.5, 139.2, 131.9, 130.7, 129.2, 129.1, 128.8 (d, *J* = 6.9 Hz), 128.2, 126.8, 123.4, 116.6, 115.7, 115.3 (d, *J* = 21.6 Hz), 114.8, 111.8, 111.5, 110.2, 108.5, 56.1, 55.9, 51.0, 31.9, 15.5; ^19^F NMR (377 MHz, DMSO-*d_6_*) δ -74.3, -114.3; HR-ESI-MS (m/z)—calculated 774.2505 [M+H]^+^, found 774.2515 [M+H]^+^; HPLC *t_R_* = 9.13 min, purity 94.3%.

### 3.2. Biological Assays

#### 3.2.1. NanoBRET™ Assay

The NanoBRET™ TE intracellular JNK3 assay was performed as previously described by Andreev et al. [27], with minor modifications. 

Cells were treated with trypsin and centrifuged (210× *g*, 5 min) one day prior to the experiment. Afterwards, cells were resuspended in Leibovitz’ L-15 medium, supplemented with 5% FCS and 10 mM HEPES, and the suspension was adjusted to a density of 300.000 cells per mL. Meanwhile the transfection reagent was prepared. For a 96-well plate, transfection carrier DNA (4 µg) (Promega, Fitchburg, WI, USA) and JNK3-NanoLuc^®^ Fusion vector (0.45 µg) (Promega) were diluted in Leibovitz’ L-15 medium (450 µL) and, finally, X-tremeGENE HP (13.5 µL) (Roche Diagnostics, Mannheim, Germany) was added. The mixture was incubated for 20 min at room temperature. Subsequently, the lipid–DNA complex was added to 9 mL of the cell suspension. A total of 80 µL of this cell suspension were added to each well of a white 96-well plate (Brand, Wertheim, Germany), which was incubated for 24 h at 37 °C (no additional CO_2_). 

The fluorescent tracer K-10 (Promega, Mannheim, Germany) was diluted in DMSO to a concentration of 25 µM (100-fold more concentrated than the final assay concentration). This was further diluted 10-fold using the tracer dilution buffer (Promega, Mannheim, Germany), yielding a dilution which was 10-fold more concentrated than the final assay concentration. Next, 10 µL of the final fluorescent tracer dilution were added to the cells (the final concentration of K-10 in the assay was 0.25 µM). 

For photocleavage experiments, each well was irradiated by UV light (365 nm) for 8 min subsequent to the addition of the tracer and the compound. As determined by a crystal violet assay (for details see Supplementary Materials) [28], the irradiation with light of a 365 nm wavelength for 8 min does not affect cell viability (Figure S2, Supplementary Materials).

For kinetic analyses experiments, plates were coated with crosslinked gelatin to improve cell adhesion. The transfection of the cells was carried out as described above, but 90 µL of cell suspension were seeded. The next day, cells were first incubated with the test compound in a 10-fold concentration of the respective IC_50_ value. After 2 h, the unbound compound was removed by washing the cells four times with 100 µL of Leibovitz’ L-15. Furimazine was then added according to the manufacturer`s protocol. Afterwards, a near-saturating concentration of tracer (1 µM) was added, and the measurement was started immediately. 

#### 3.2.2. Point Mutation of Cys154 in the JNK3-NanoLuc Fusion Vector

To generate the cDNA of the JNK3(C154A)-NLuc mutant, JNK3-NanoLuc^®^ Fusion vector (Promega) was used as a template, and two partially overlapping primers (5′-GCCAACTTAGCTCAAGTGATTCAGATGGAATTAG and 5′-CACTTGAGCTAAGTTGGCATCCATCAGTTC) (synthesized by Eurofins, Ebersberg) annealed to the template. Phusion HF DNA Polymerase was obtained from New England BioLabs (Frankfurt, Germany). The polymerase chain reaction (PCR) was carried out as described in the Phusion HF polymerase protocol. The PCR product was treated with Dpn I endonuclease and transformed into competent E. coli Top 10 cells. Point mutation was confirmed by sequencing (Eurofins Discovery). 

## Figures and Tables

**Table 1:** Structures and biological activities of compounds **1**, **8**, **10**, **13**, **14**, and **17**.

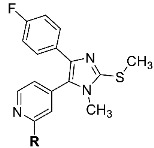			
Cpd.	R	JNK3 IC_50_ [nM] ^a^	JNK3 IC_50_ ± SEM [nM] ^b^
**1**	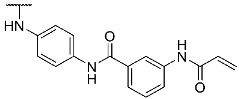	6	243 ± 75
**8**	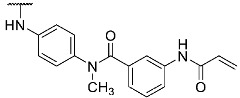	14	1360 ± 342
**10**	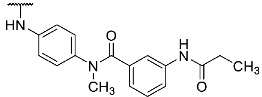	24	5015 ± 97
**13**	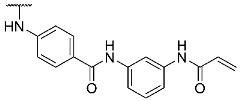	13	1064 ± 252
**14**	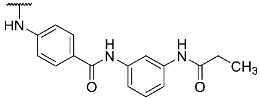	22	>10,000
**17**	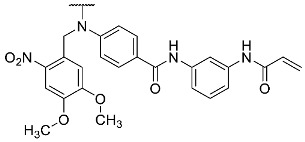	n.d. ^c^	9487 ± 538

^a^ IC_50_ values were determined in a radiometric JNK3 activity assay at Eurofins Discovery; ^b^ IC_50_ values were determined in a NanoBRET^TM^ TE intracellular JNK3 assay and are the means of at least two independent experiments; ^c^ n.d., not determined.

## Data Availability

Data is contained within the article and supplementary material.

## Supplementary Materials

The following supporting information can be downloaded at: https://www.mdpi.com/article/10.3390/ph16020264/s1, Scheme S1: Synthetic pathway toward pyridinylimidazole **7**; experimental protocol for the glutathione stability assay including Figure S1: Evaluation of the chemical stability of compounds **8**, **13** and **JNK-IN-8**; experimental protocol for the crystal violet assay including Figure S2: Crystal violet assay for determination of the cytocidal effect of UV irradiation (365 nm, 8 min).
