# Supplementary material for: Photocaging of Pyridinylimidazole-Based Covalent JNK3 Inhibitors Affords Spatiotemporal Control of the Binding Affinity in Live Cells
Source: Pharmaceuticals (Basel). 2023 Feb 9;16(2):264. doi: 10.3390/ph16020264 (PMC9962329; doi:10.3390/ph16020264)
Supplement: Supplementary file 1 [file pharmaceuticals-16-00264-s001.zip › pharmaceuticals-2145021-supplementary.pdf]

Supplementary Materials:

Article

Photocaging of Pyridinylimidazole Based Covalent JNK3 Inhibitors Affords Spatiotemporal Control of the Binding Affinity in Live Cells

Beate Sandra Hoffelner [1], Stanislav Andreev [1], Nicole Plank [1] and Pierre Koch [1,*]

[1] Department of Pharmaceutical/Medicinal Chemistry II, Institute of Pharmacy, University of Regensburg, Universitätsstraße 31, 93053 Regensburg, Germany.

* Correspondence: pierre.koch@chemie.uni-regensburg.de; Tel.: +49 941 943 4827

Table of Contents

1. Synthetic Pathway Toward Pyridinylimidazole **7**.	**S2**
2. Glutathione Stability Assay.	**S3**
3. Crystal Violet Assay.	**S4**
4. References	**S4**

1. Synthetic Pathway Toward Pyridinylimidazole **7**

Reported synthetic approach towards 4-(4-(4-fluorophenyl)-1-methyl-2-(methylthio)-1*H*-imidazol-5-yl)pyridin-2-amine (**7**). [1,2]

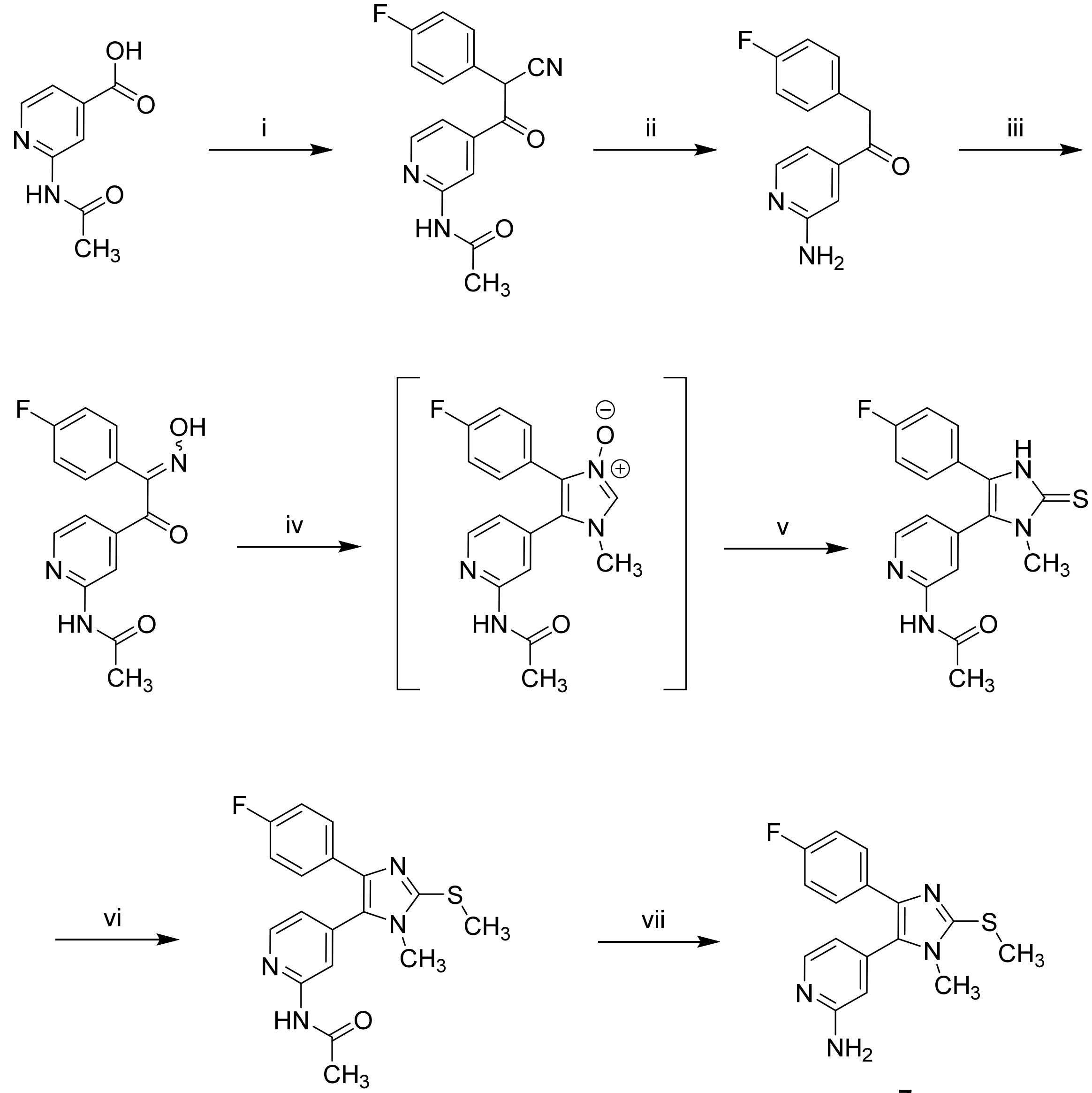

7

Scheme S1. Synthetic pathway toward pyridinylimidazole **7**. Reagents and conditions: i) 1. Carbonyldiimidazole, DMF, r.t., 2. 4-fluorophenylacetonitrile, K*t*BuO, DMF, 120 °C; ii) 48% aq. HBr, reflux temperature; iii) 1. acetic anhydride, 4-(dimethylamino)pyridine, reflux temperature, 2. isoamyl nitrite, $NaOCH_3$, MeOH, r.t.; (iv) 1,3,5-trimethylhexahydro-1,3,5-triazine, ethanol, reflux temperature; (v) 2,2,4,4-tetramethylcyclobutane-1,3-dithione, DCM, r.t.; (vi) iodomethane, K_2CO_3, methanol, r.t.; (vii) 10% aq. HCl, reflux temperature.

2. Glutathione Stability Assay

The general reactivity of compounds **8**, **13** and **JNK-IN-8** towards nucleophiles was assessed by a glutathione stability assay.[3] 5 µL of a 10 mM DMSO solution of the respective compound were diluted with 590 µL of HPLC grade acetonitrile. 5 µL of a 4 mM DMSO solution of Promethazine hydrochloride were added as internal standard and the mixtures further diluted with 400 µL of PBS buffer (pH = 7.4). 500 µL of each compound solution were mixed with 500 µL of a 10 mM glutathione solution in PBS buffer resulting in a final compound concentration of 25 µM and a final glutathione concentration of 5 mM (200-fold excess). The reaction mixtures were incubated at 37 °C for 3 days and monitored at several timepoints by HPLC utilizing the method described in chapter 3.1.1 with an injection volume of 50 µL. The experiments were carried out in duplicates. Compound degradation was calculated based on the decreasing area under the curve (AUC) relative to the internal standard and plotted against the experiment time (Figure S1).

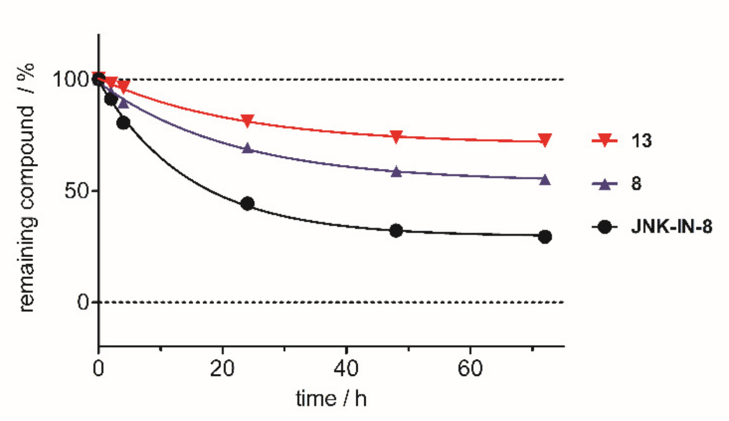

Figure S1. Evaluation of the chemical stability of compounds **8**, **13** and **JNK-IN-8** in the glutathione stability assay.

3. Crystal Violet Assay

The cytocidal effect of the UV light was determined by a crystal violet assay.[4] HEK293T cells were transfected and seeded as described in section 3.2.1. The next day, 20 µl of Leibovitz' L-15 medium supplemented with 5% FCS and 10 mM HEPES was added to each well. Subsequently, the cells in one plate were irradiated with UV light (365 nm) for 8 minutes, whereas a second plate served as a control. After incubating the plates for 2 h, the medium was discarded and cells were first fixed for 20 min with 2% glutardialdehyde in PBS (100 µL/well) and then stained with 0.02% crystal violet (100 µL/well) for 20 min. Afterwards, excess dye was removed by washing the plates with water. The dye bound by the cells was redissolved with 70% EtOH (200 µl/well). The absorbance (580 nm) was measured by an EnSpire plate reader (PerkinElmer, Rodgau, Germany) (Figure S2).

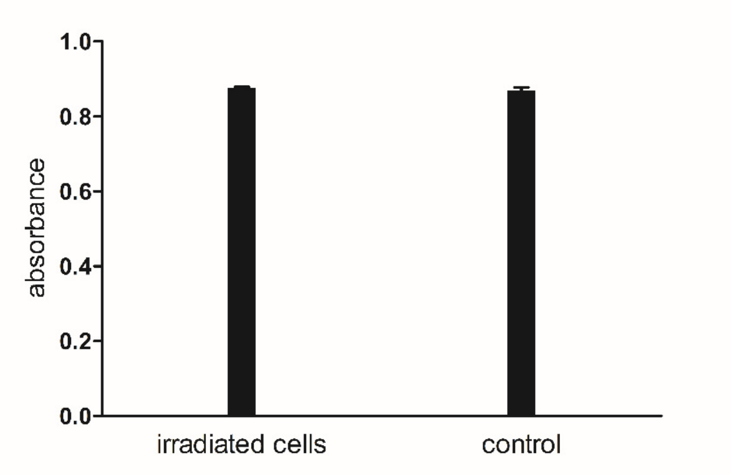

Figure S2. Crystal violet assay for determination of the cytocidal effect of UV irradiation (365 nm, 8 minutes).

4. References

1. Laufer, S.A.; Zimmermann, W.; Ruff, K.J. Tetrasubstituted imidazole inhibitors of cytokine release: Probing substituents in the N-1 position. *J. Med. Chem.* **2004**, *47*, 6311-6325, doi:10.1021/jm0496584.
2. Wagner, G.K.; Kotschenreuther, D.; Zimmermann, W.; Laufer, S.A. Identification of regioisomers in a series of N-substituted pyridin-4-yl imidazole derivatives by regiospecific synthesis, GC/MS, and H-1 NMR. *J. Org. Chem.* **2003**, *68*, 4527-4530, doi:10.1021/jo026619w.
3. Forster, M.; Liang, X.J.; Schröder, M.; Gerstenecker, S.; Chaikuad, A.; Knapp, S.; Laufer, S.; Gehringer, M. Discovery of a Novel Class of Covalent Dual Inhibitors Targeting the Protein Kinases BMX and BTK. *Int. J. Mol. Sci.* **2020**, *21*, doi:10.3390/ijms21239269.
4. Bernhardt, G.; Reile, H.; Birnbock, H.; Spruss, T.; Schoenenberger, H. Standardized Kinetic Microassay to Quantify Differential Chemosensitivity on the Basis of Proliferative Activity. *J. Cancer Res. Clin.* **1992**, *118*, 35-43, doi:10.1007/Bf01192309.
